# Construction of VGG16 Convolution Neural Network (VGG16_CNN) Classifier with NestNet-Based Segmentation Paradigm for Brain Metastasis Classification

**DOI:** 10.3390/s22208076

**Published:** 2022-10-21

**Authors:** Abdulaziz Alshammari

**Affiliations:** College of Computer and Information Sciences, Imam Mohammad Ibn Saud Islamic University (IMSIU), Riyadh 11432, Saudi Arabia; aashammari@imamu.edu.sa

**Keywords:** brain metastases, classification, segmentation, deep learning, neural network, VGG16_CNN, NestNet

## Abstract

Brain metastases (BMs) happen often in patients with metastatic cancer (MC), requiring initial and precise diagnosis of BMs, which remains important for medical care preparation and radiotherapy prognostication. Nevertheless, the susceptibility of automated BM (ABMS) diagnosis is unfairly great for minute BMs, and integrating into medical exercises to distinguish true metastases (MtS) from false positives remains difficult. For enhancing BM classification execution, MtS localization is performed through the NestNet framework. Subsequent to segmentation, classification is performed by employing the VGG16 convolution neural network. A novel loss function is computed by employing the weighted softmax function (WSF) for enhancing minute MtS diagnosis and for calibrating susceptibility and particularity. The aim of this study was to merge temporal prior data for ABMS detection. The proffered VGG16_CNN is capable of differentiating positive MtS among MtS candidates with high confidence, which typically needs distinct specialist analysis or additional investigation, remaining specifically apt for specialist reinforcement in actual medical practice. The proffered VGG16_CNN framework can be correlated with three advanced methodologies (moU-Net, DSNet, and U-Net) concerning diverse criteria. It was observed that the proffered VGG16_CNN attained 93.74% accuracy, 92% precision, 92.1% recall, and 67.08% F1-score.

## 1. Introduction

Patients with MC possess an elevated threat of acquiring brain metastases (BMs), having a rough occurrence rate of up to 40%. The advancement of BM causes decreased or nil efficiency of general systemic medical care [1]. Hence, victorious medical care of BMs remains extremely important for a patient’s existence and standard of living. Since whole-brain radiation therapy (WBR) of BMs leads to cognitive deteriorations, stereotactic radiosurgery (SRSy) is attracting increased interest for BM therapy. SRSy dispenses highly focused radiation toward the metastasis areas with less dosage toward the neighboring typical brain tissues. Therefore, this leads to not many side-effects when compared with WBR [2]. For SRSy therapy preparing and scheduling, the BM count, dimension, edge, and position remain significant data that need precise identification for ensuing BM segmentation. Presently, BMs can be detected physically by neuroradiologists and radiation oncologists through a time-consuming process that suffers from inter-rater variability [3]. In particular, some metastases (MtS) are simply disregarded in physical identification since they are positioned only in some image slices and normally possess low contrast. Furthermore, a few anatomical forms such as blood vessels resemble BMs in two-dimensional intersection planes, hindering their detection [4]. Hence, computer-aided automated brain metastasis (ABMS) detection possesses significant medical value. For ABMS identification and segmentation, standard machine learning methodologies such as template matching, support vector machine, and AdaBoost have been implemented [5]. Nevertheless, these proved mediocre compared to the recent deep learning (DL) methodologies.

Owing to the latest escalation of DL approaches, even though most analysts concentrated on the segmentation of chief brain tumors (BTs) such as gliomas, the study of DL for BM identification and segmentation is improving. Concerning neural network (NN) frameworks for BM segmentation, three-dimensional U-Net and DeepMedic1 remain the preferred networks [6]. Other NNs include GoogLeNet, V-Net, faster R-CNN, single-shot detectors, and custom convolutional NNs (CNNs) [7]. For saving memory, the GoogLeNet technique employs seven slices: a center slice with six nearby slices, resembling a 2.5-dimensional paradigm. The remaining NNs employ three-dimensional sub-volumes for training [8]. To train segmenting NNs, loss functions (LFs) such as binary cross-entropy (BCE), Dice similarity coefficient (DSC), and intersection over union (IOU) are normally implemented. For enhancing BM segmentation, novel LFs can be proffered. Herein, the contribution of this process is ensuring that the network employs its whole receptive field. While conducting training, the extra outputs are employed as supplementary loss layers by correlating them to inferior variants of the reference label data. The aim of this study was to differentiate true positive (TP) MtS among MtS candidates (MCs) with high confidence, which typically requires unique specialist analysis or additional investigation, remaining specifically apt for specialist reinforcement in actual medical exercise. VGG16_CNN attains great susceptibility for BM identification and differentiates MtS among MCs with high confidence. 

The remainder of this paper is structured as follows: Section 2 highlights some existing studies, Section 3 presents the background of prediction criteria for BM classification, Section 4 describes the proffered technique and methods, Section 5 presents the experimental results and discussion, and Section 6 provides a conclusion and insights for future study.

## 2. Associated Studies

Tumors and neoplasms are created when cells in any part of the body develop abnormally and form a mass. There are two different kinds of tumors: one is benign and the other is cancerous or “malignant”. A benign tumor does not represent a high danger to the body because it does not spread to other parts of the body. A malignant tumor, however, is extremely dangerous as it spreads to other body organs [9]. This section addresses the associated literature and conveys the limitations addressed by the proffered methodology. Generally, similar to this study, many studies concentrated upon diverse kinds of MtS classification.

The authors of [10] introduced a new three-dimensional multi-attention guided multitask learning network for concurrent gastric tumor segmentation with lung nodule classification that achieves complete utilization of the corresponding data with disparate sizes, scales, and tasks. In particular, the task correspondence and heterogeneity of the CNN comprise scale-aware attention-guided shared feature learning (AGSFL) for refinement and global multiscale (MS) features, and task-aware AGSFL for task-particular discriminatory features. The major applications of convolutional neural networks are image recognition and classification. It is also the only use case involving the most advanced frameworks in medical imaging.

The authors of [11] proposed a few-shot learning methodology for classifying an image patch that comprises tumor cells. Specifically, a patch-level unadministered cell scoring technique is presented that solely depends upon images having restricted labels. The chief notion of this methodology remains that, while clipping patch A from whole-slide images (WSI), in addition to a sub-patch B from patch A, A’s cell count remains constantly above that of B. Accordingly, the unlabeled images can be employed for learning the cell counting data for extracting notable features. 

The authors of [12] presented a new deep regional MtS segmentation (DRMS) architecture for lymph node status classification. Initially, a deep segmentation network (DSN) was proffered for identifying the regional MtS within the patch level. Next, the density-based spatial clustering of applications with noise (DBSCAN) was embraced for prognosticating the complete MtS from independent slides. Lastly, pN phases in patients were decided by totaling independent slide-level prognoses.

The moU-Net performed well for the segmentation of glioblastoma and low-rank glioma [13]. With similar network encoder and decoder layers to the conventional U-Net, moU-Net includes an extra output layer toward the decoder. U-Net is marked by an encoder that extracts lower-grade portrayals of the input data (ID) and is associated with a decoder that rebuilds a correlated label map with skip connections between intermediate phases of the two units. Since its initial proposal, many augmentations concerning the network framework and the training procedure have been applied to U-Net [14].

The authors of [15] computed the susceptibility and particularity of the paradigm for each pel, as opposed to lesion, greatly benefiting bigger lesions. The utilized database also possesses a common dispensation of primary cancer (PC) kinds that are employed for exhibiting the DCNN’s strong execution for disparate chief tumors. The authors were certain that their work remained a rational and, hence, medically pertinent observance of DCNN’s anticipated execution. 

The ideal therapy of patients with BMs relies upon the intended condition. Frequent operation, radiation operation, whole-brain (WH) radiation therapy, and chemotherapy can be employed together for attaining lengthier remissions and ideal symptom relief. Patients with several MtS generally obtain WB irradiation (WBI). It remains certainly arduous to seek the MtS degree using the aforementioned methodologies. Thus, a new classifier is proffered in this study.

In spite of encouraging classification findings in many evaluated deep learning studies, care is urged because overfitting must be avoided when using big, annotated, and good datasets [16]. Three studies [17,18,19] tested ensemble learning techniques. In order to create a model that outperforms individual classifiers, ensemble learning combines many algorithms that are trained on either various datasets or the same data, but with distinct algorithms.

## 3. Background of Predictive Attributes for BM Classification

The prognosis of patients with BMs is normally poor and notably ruins quality of life. The median survival of patients with BMs does not exceed 5 months in spite of the present therapeutic possibilities. However, the description of perfectly identified predictive subsets remain vital for the selection of a customized treatment plan. It remains significant to detect subsets of patients with suitable predictive attributes, which can then lead to the development of therapy targeting survival and an improved quality of life [20]. Conversely, for patients devoid of ideal extracranial illness control and/or comorbidities (which can restrict the tolerance of fierce therapies), the therapy goal remains to stabilize BMs for administering symptoms and restricting malignancy. 

Significant predictive attributes include key performance status (KPS), the BM count, the lack of systemic MtS, principal tumor control, and age. The Radiotherapy Oncology Group (RTOG) modeled a prediction ranking scheme inferred from the assessment of individual predictive attributes for patients with BMs and proposed three subsets (RPA classes) [21]. This led to the establishment of a novel and elaborate predictive index (Graded Prognostic Assessment, GPA), which considers four factors: age, KPS, the existence of extracerebral MtS, and BM count [22].

## 4. System Paradigm

**Figure 1:**
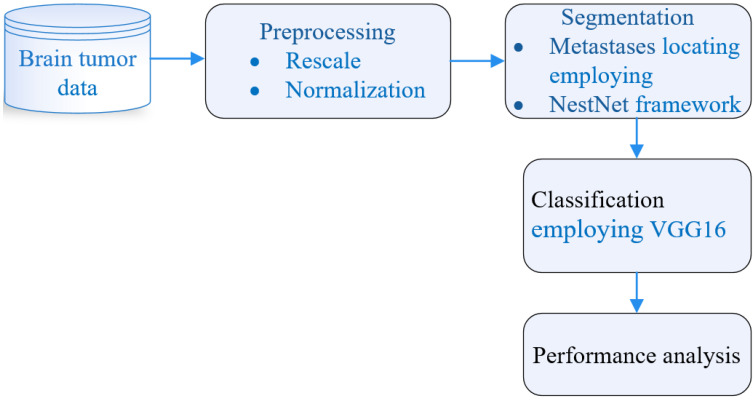
Comprehensive framework for BM classification.

A comprehensive schematic of the proposed BM classification employing the BT database is illustrated in Figure 1. The BT dataset comprised 3064 T1-weighted contrast-optimized images. The database was then preprocessed, rescaled, and generalized. Next, segmentation was performed and MtS identification was carried out by employing the NestNet (NtNt) framework. Subsequent to segmentation, classification was performed by employing the VGG16 convolution NN. 

### 4.1. Image Initialization

The image data were retroactively recovered from our institutionalized e-clinical registers and anonymized prior to the assessment. Axial three-dimensional spoiled gradient recall or magnetization-prepared rapid gradient echo T1-weighted contrast-augmented MR images were recovered for assessment. Patients with single or multiple BMs were chosen consecutively while conducting the medical readout. MRI was performed using 1.5 T and 3 T scanners from two chief merchants. Overall, data were recovered for 121 patients with 2053 MtS, resulting in 14,350 image slices from a total of 361 scans. Patients were incorporated into the research for definitive medical and imaging detection of BMs. Patients with ambiguous lesions were not considered. Pathological verification of BMs through brain biopsy or resection was performed in 43 patients (36%). In the remaining 78 patients (64%), brain operation was not performed; thus, pathologic detection using brain tissue was unavailable. However, as the doctors did not approve the risk of brain surgery in consideration of the BMs identified through medical and imaging data, all 78 patients possessed a noted detection of PC elsewhere in the body. Accordingly, the team unanimously agreed to the medical detection of BMs in these patients. The majority of patients completed several scans, whereas some completed a single scan.

### 4.2. Brain Image Preprocessing

Prior to employing images for training or testing, a sequence of procedures was applied for image preprocessing. Firstly, since the initial images had disparate matrix dimensions, the images were padded with zeroes. Scaling was carried out next, if required, for transforming entire images to achieve similar matrix dimensions of 900 × 900. For the reference image (RI), the histogram consisted of homogeneous low-intensity regions of interest (ROIs) (low-intensity region—LIR) and high-intensity ROIs (high-intensity region—HIR). The histogram ranged from LIR to HIR illumination levels. The image intensities were mapped as values between HIR and LIR; the former was considered the value at the maximal decile, while the latter was considered the value at the minimal decile. This assisted in eliminating background noise and outliers as defined above according to the following equation:

(1) $$f^{\prime }\left(x,y,z\right)=\frac{f\left(x,y,z\right)-LIR}{HIR-LIR},$$

where f(x,y,z) indicates the initial reference gray value at (x,y,z), and $f^{\prime }\left(x,y,z\right)$ indicates the corresponding transitioned grayscale value. Subsequent to primary scaling, histogram normalization can be performed. The ROI histogram is elongated and transformed to cover all grayscale levels (GL) within the input image (IpI) as follows:

(2) $$g^{\prime }\left(x,y,z\right)=\frac{HIR-LIR}{S_{max}-S_{min}}(g\left(x,y,z\right)-S_{min}+LIR.$$

When the IpI’s target histogram g(x, y, z) begins at $S_{min}$ and elongates to $S_{max}$ GL within the ROI, the image can be scaled between the bottom edge $m_{1}^{\prime }\;$and the top edge $m_{2}^{\prime },\;$whereby the voxels within the novel normalized image g′(x, y, z) are between a minimal degree (LIR) and a maximal degree (HIR). The resulting variables $m_{1}$ and $m_{2}$ remain the bottom edge and top edge of the ROI before scaling up. The normalization action is expressed as N(x, y, z) according to the following equation:

(3) $$N\left(x,\;y,\;z\right)=\{\begin{array}{c} \mu_{s}+\left(g\left(x,y,z\right)-\mu_{i}\right)\frac{LIR-\mu_{s}}{S_{1i}-\mu_{i}},m_{1}^{\prime }\leq g\left(x,y,z\right)\leq \mu_{i}\;\\ \mu_{s}+\left(g\left(x,y,z\right)-\mu_{i}\right)\frac{HIR-\mu_{s}}{S_{2i}-\mu_{i}},\mu_{i}\leq g\left(x,y,z\right)\leq m_{2}^{\prime } \end{array},.\;$$

where $\mu_{i}$ and $\mu_{s}$ represent the average values for the IpI and ROI histograms, respectively. $S_{1i}$ and $S_{2i}$ represent the voxel values of the IpI. 

### 4.3. MtS Localization Employing NtNt Framework

The preprocessed images incorporated the following localization-related features:

- Left/right hemisphere or central form,
- Cerebral lobes: frontal, parietal, temporal, and occipital lobe,
- Insular cortex,
- Subcortical forms: basal ganglia, thalamus, brainstem, corpus callosum, and cerebellum,
- Eloquent brain regions: vision center (the region surrounding Sulcus calcarinus), auditory center (Gyri temporales transverse), Wernicke’s region (from Gyrus temporalis superior’s dorsal area to the parietal lobe’s Gyri angularis et supramarginal), Broca’s region (Gyrus frontalis inferior’s Pars triangularis et opercularis), the primary somatosensory cortex (Gyrus postcentralis), and primary somatomotor cortex (Gyrus praecentralis).

The localization-related features were not considered as cooperatively unique, whereby each lesion was ascribed to several of the abovementioned classes, e.g., left hemisphere, temporal lobe, and vision center.

Pairwise differences (PDs) were calculated for each lesion’s binary feature vectors (FVs), as well as histological subtypes, by exploring the Jaccard distance = (1 − Jaccard index). The consequential distance matrix (DM) (size 239 × 239) was clustered by employing agglomerative stratified clustering with mean linkage. Additionally, PDs were encapsulated over entire lesions (by taking the mean) within a histological tumor type to yield the DM for each type. The resulting DM was transitioned into an undirected graph’s (UG) affinity matrix by calculating its entries ai,j=1−di,j with di,j remaining the DM’s (i,j)-th entry. Lastly, the UG could be viewed by employing a spectral layout with high-affinity nodes closer than low-affinity nodes. 

### 4.4. MtS Area Segmentation Employing NtNt Framework

To effectively perform image segmentation, this study proposes a novel DL method consisting of an encoding module (EU) in which two disparate timespans can be employed as the input for executing feature extraction (FE). This unit contains four stratified ranks, and feature tensors in a similar rank contain a similar length and breadth. Nevertheless, the channels remain disparate. The method also consists of a decoding module which segments the extracted features and upsamples them toward an image with similar length and breadth to the change-labeled image. The NtNt framework consists of two corresponding units for processing images of disparate timespan. 

**Figure 2:**
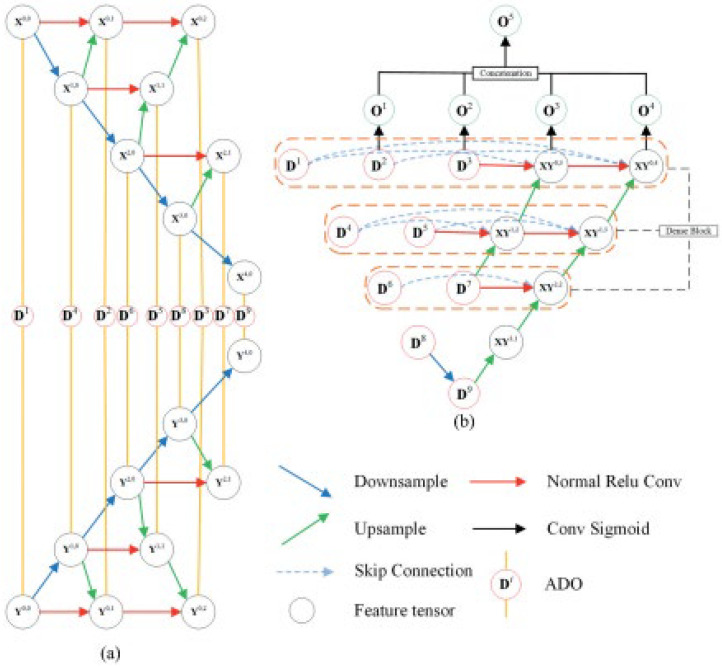
Segmentation procedure employing NtNt framework.

The segmentation procedure framework is illustrated in Figure 2, where Figure 2a depicts the downsampling module (DM) [23]. Various timespan images can be employed as the ID for this DM. Subsequent vectors within the FV of a similar level can be attained from the previous vector (PV) following three procedures: ReLU, Conv (red arrows), and splicing of entire PVs and next-level vectors (NLVs) after upsampling (green arrows). NLVs can be downsampled from the previous-level vector (PLV; blue arrow). Figure 2b depicts the upsampling module. A dense framework can be employed for the top three layers, and various level vectors can be linked via the upsampling procedure. Here, four outcomes from O1 to O4 can be ultimately attained, which are later joined using a concatenating procedure and transitioned to O5 via a convolution procedure.

### 4.5. Encoding Module

The feature extraction units target multiscale convolutional features in images X and Y. $F_{x/y}^{i,j}$ is employed to differentiate the features from X and Y. $F_{x/y}^{i,j}$ is computed using Equation (4).

(4) $$F_{x/y}^{i,j}=c\left(\alpha \left(F_{\frac{x}{y}}^{i,j},\;u\;F_{\frac{x}{y}}^{i+1,j+1}\right)\right).$$

Considering j∈{(0,1),(0,2),(1,1),(2,1)} and $F_{x/y}^{i,j}=d\left(F_{\frac{x}{y}}^{i-1,j}\right)$, j∈{1,0,(2,0),(,0),(4,0)}. In Equation (4), $F_{x/y}^{i,j}$ and $F_{x/y}^{i,j}$ portray the features from images X and Y, c(.) indicates the concat procedure, which concatenates the features by channels, u(.) indicates the upsample procedure, which upsamples the feature’s length and breadth to half of the initial dimension, α (.) indicates the EM’s calculation, and x/y indicates X or Y. ADO is employed for synthesizing the features from images X and Y. $F_{x/y}^{i,j}$, as the ADO’s outcome, can be computed using Equation (5).

(5) $$F_{D}^{i}=\beta \left(F_{x}^{a,b},F_{y}^{a,b}\right).$$

Considering i ∈1,2,3…9, $F_{x}^{a,b}$ and $F_{y}^{a,b}$ portray the features from images X and Y. β(.) denotes the ADO described in Equation (1). The EM remains a dual framework, in which two alike units are employed for feature extraction, feature downsampling, eigenvalue activation, and stitching of the disparate levels of feature layer on the bitemporal images. The feature data from two images can be extracted by EM. 

### 4.6. Decoding Module

The outcomes of the initial elements $\left(D^{1}-D^{9}\right)$ are considered as the downsampling module input. In the DM, similar level features are linked to the remaining features, and low-level features are upsampled and concatenated to the previous level’s features. $F_{x,y}^{i,j}$ is employed to portray the value of $XY^{i,j},\;$where $F_{x,y}^{i,j}$ is computed as follows:

(6) $$F_{x,y}^{i,j}=c(u\left(F_{x,y}^{a,b}\right),F_{D}^{t}.$$

A dense block unit is employed in the downsampling module’s top three layers for feature fusion to enhance the effectiveness of feature usage. For each layer, the feature maps of the previous layer are employed as the input. In line with the remaining transition identification network units, the NtNt framework’s chief disparities incorporate the sima network to execute segmentation of two individual images. To resolve the issue where the transformed and untransformed pels remain unbalanced, the LF employed in this study comprises balanced binary cross-entropy loss (BBCEL) and Dice loss. The entire layer result at level 0 computes the loss with a disparate weight. The comprehensive LF is described as

(7) $$L=\overset{s}{\underset{i=1}{\sum }}\omega_{i}L_{side}^{i},$$

where $\omega_{i}$ portrays the i-th output layer’s weight. $L_{side}^{i}$ comprises BBCEL and DL, which can be described by Equation (8).

(8) $$L_{side}^{i}=L_{BCE}^{i}+L_{dice}^{i}.$$

Regardless of the training database or confirmation database, most images only possess a tiny area that remains transformed. A few LFs employed in segmentation do not perform well in transition identification. Thus, the balanced BBCEL is considered in the LF portion, which can be described by Equation (9).

(9) $$L_{BCE}=\overset{N}{\underset{n=1}{\sum }}w_{n}[y_{n}\times logx_{n}+\left(1-y_{n}\right)\times log\left(1-x_{n}\right)],$$

where $y_{n}$ indicates the ground truth of pel n (zero or one), and $x_{n}$ indicates the estimated value of pel n. $w_{n}$ portrays the ratio of the present pel (zero or one) to the sum of pels. N is the sum of pels.

### 4.7. Classification Employing VGG16 Conv NN

In Dice loss, the CNN or ConvNet denotes the deep NN commonly implemented for assessing an image’s visual qualities. VGG-16 is a CNN mostly employed to process images. VGG-16′s 16 layers consist of convolutional, pooling, and fully connected layers (FCLs).

**Figure 3:**
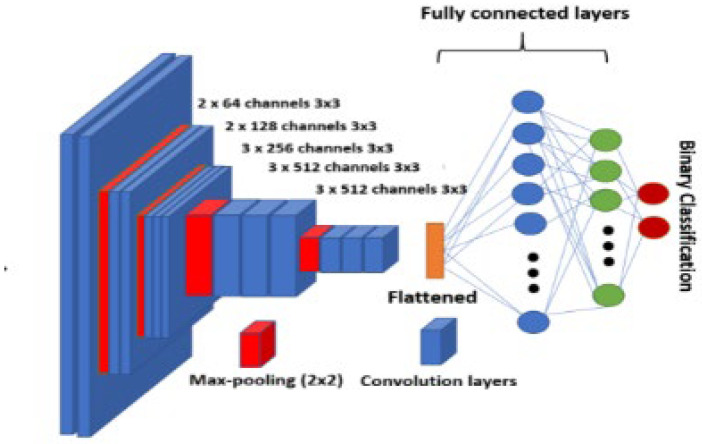
VGG-16 CNN framework.

Figure 3 illustrates the VGG-16 CNN framework. The network’s framework contains multiple convolution layers (CLs) and three FCLs. These FCLs are portrayed as dense layers [24]. Training images are used as input. Patches of these images are extracted and forwarded to the network during training.

### 4.8. Shared Core Layer

The multitask network of this study was constructed using the VGG16 network proposed by Simonyan and Losch [25], constituting a stack of 3×3 CLs (Conv) with nonlinearity (ReLU) and 3×3 max-pooling layers (MPL) (pool) for downsampling. The framework is reiterated until the result contains a tiny spatial dimension, and a decision is made with respect to the result by the FCLs and the softmax layer to compute class probabilities. As this network consists of five MPLs, the spatial dimension is lessened by a factor of 25 or 32 prior to making it to the FCLs.

### 4.9. Atrous Convolution Block

The atrous convolution (AC) block classifies the ID into four divisions for corresponding computation. The four divisions employ a similar quantity of convolution kernels (CKs). The initial division employs a conventional 3 × 3 convolution. The next division employs an AC with a CK dimension of 3 × 3 and two dilation rates (DRs). The next division employs an AC with a CK dimension of 3 × 3 and three dilation rates. The last division employs an AC with a CK dimension of 3 × 3 and five DRs. Following the four CLs for classification into divisions, there is a transformed linear module (Relu) and a batch normalization (BN) procedure. Lastly, the concat procedure is employed to concatenate the feature maps of the four divisions. AC can efficiently enlarge the receptive region without affecting computation. The AC block employs convolutions with various dilation rates to concurrently extract images at several scales, facilitating network FE.

### 4.10. Optimized Boosting Strategy

The boosting strategy optimizes the precision at the start of augmenting the number of classifiers. If a great number of weak classifiers (WC) is employed, this would not continually enhance the precision and would result in overfitting [26]. In this study, exit was observed that three WCs were adequate for creating a robust classifier that considerably enhances the classification’s execution. One-third of the data samples were randomly chosen for training the initial WC VGG16-T. Considering misclassified samples from the previous round as the training data (TD), the increasing weight of erroneous samples could be improved according to criteria for training the subsequent WC VGG16-T to learn many portrayal features, thereby alleviate the unbalance. Many characteristic and discriminatory samples can be chosen for training and testing to augment the method’s strength.

**Figure 4:**
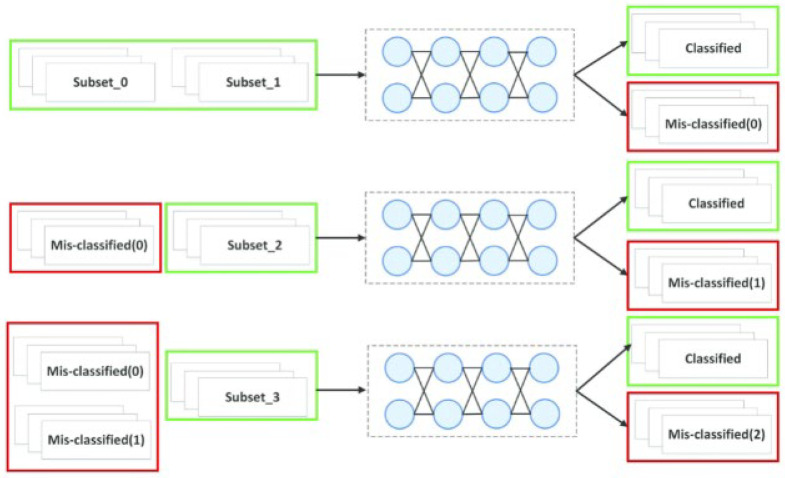
Weighted boosting methodology’s framework.

As shown in Figure 4, the chief WC was trained by the filtered databases [27]. The initial error data (misclassified (0)) within the red rectangle box and the novel training data were fused for training the next WC. The below criteria were enhanced by employing erroneous data from the initial two data and the remaining novel data. To decrease the number of false positives (FPs), boosting is an analytical learning methodology commonly employed with optimized classifiers. The classifiers remain linearly joined to enhance classification by assigning disparate weights to training samples (TS).

### 4.11. Weighted Softmax Function

The softmax classifier (SC) is a normalization method that splits target variables into multiple classes [28,29]. SC was employed for BM pathological feature identification in CT images.

It can be presumed that there are N IpIs ${\left\{x_{i},y_{i}\right\}}_{i=1}^{N}$ in every image according to a tag out of $\left\{y_{i}\in \left\{1,2,3,\ldots .k\right\},k>2\right\}$. The sum of classes is indicated by k (*k* = 4 in this methodology). For a provided test image $x_{i}$, the hypothesis function is to predict the probability value $p(y_{i}=j/x_{i)}$ of every class *j*. The value of $h_{\theta }\left(x_{i}\right)$ can be computed as follows:

(10) $$h_{\theta }\left(x_{i}\right)=\left[\begin{array}{ccc} p\left(y_{i}=1\right) & \cdots & x_{i};\theta \\ \vdots & \ddots & \vdots \\ p\left(y_{i}=k\right) & \cdots & x_{i};\theta \end{array}\right]=\frac{1}{\sum_{j=1}^{k}\exp\left(\theta_{j}^{T}\right)},$$

where $\frac{1}{\sum_{j=1}^{k}\exp\left(\theta_{j}^{T}\right)}$ portrays the probability dispensation’s normalization, i.e., the sum of entire probabilities remains 1, and θ portrays the criteria. The SC’s LF J(x,y,θ) can be expressed as

(11) $$J\left(x,y,\theta \right)=\frac{1}{N}\{\overset{N}{\underset{I=1}{\sum }}\overset{K}{\underset{J=1}{\sum }}1\{y_{i}=j\}\}1b\frac{\exp\left(\theta_{j}^{T}x_{i}\right)}{\sum_{j=1}^{k}\exp\left(\theta_{j}^{T}x_{i}\right)},$$

where $1\{y_{i}=j\}=\{\begin{array}{c} 0,\;y_{i\in j}\\ 1,\;y_{i∋j} \end{array}$.

Imbalanced TSs might result in the training concentrating on classes with a great quantity of samples. The generalization capability with respect to the test data can be improved by fixing w within the softmax loss function (SLF), whereby tiny class samples (CSs) are multiplied by a large weight and large class samples are multiplied by a tiny weight to diminish the class unbalance issue within this database, thus enhancing detection precision. The weighted SLF J (x,y,θ) is calculated as follows:

(12) $$J\left(x,y,\theta \right)=\frac{1}{N}\{\overset{N}{\underset{I=1}{\sum }}\overset{K}{\underset{J=1}{\sum }}w_{i}1\{y_{i}=j\}\}1b\frac{\exp\left(\theta_{j}^{T}x_{i}\right)}{\sum_{j=1}^{k}\exp\left(\theta_{j}^{T}x_{i}\right)},$$

where $w_{i}=\frac{M_{i}}{M_{j}}$ portrays the LF’s weight, $M_{i}$ portrays the TSs, and $M_{j}$ portrays the TS class sample count. The LF can be reduced using stochastic gradient descent (SGD) methodology.

## 5. Experimental Analysis

During training, the mini-batch SGD with a batch size of 16, momentum of 0.9, and weight decay of 0.0005 was employed. The “poly” learning rate (LR) policy was utilized in which the learning rate was multiplied by a power of 0.9, and the original LR was 0.001. The maximal number of epochs was 100 [30]. Table 1 exhibits the VGG16_CNN’s weight and biases.

### 5.1. Database Explanation

Data were collected out for 233 patients with three BT types—meningioma (708 slices), glioma (1426 slices), and pituitary tumor (930 slices). Because of the limitations of the depository’s file dimensions, the complete database was divided into four subcategories and obtained in four .zip files comprising 766 slices each. Fivefold cross-validation indices were also determined. Patients underwent SRSy according to the CyberKnife stereotactic therapy scheme. For therapy arrangements, MR images incorporating contrast-enhanced T1-weighted (T1c), T2-weighted, and fluid-attenuated (FLAIR) images were considered. Patients not meeting these criteria were omitted from the research. Target forms (planning target volumes—PTVs) were physically depicted on the MR images by board-authorized neurosurgeons or radiation oncologists.

### 5.2. Execution Metrics

In this study, accuracy, precision, recall, and F1-score were chosen as the evaluation criteria. The proffered VGG16_CNN was correlated with three conventional methodologies (moU-Net, DSNet, and U-Net) centered upon the above criteria.

Accuracy indicates the capability of the comprehensive prognosis generated by the method. The true positive (*TP*) and true negative (*TN*) depict the ability to determine the existence or lack of MtS. The false positive (*FP*) and false negative (*FN*) depict cases of false prognosis by the employed method.

(13) $$Accuracy=\frac{TP+TN}{TP+TN+FP+FN}.$$

The precision rate is the proportion of positive samples to total samples with MtS.

(14) $$Precision=\frac{TP}{TP+FP}.$$

Recall reflects the capability to precisely identify MtS within the database; the susceptibility computation in no way considers indefinite test outcomes since the test can be reiterated and indefinite samples are omitted from assessment.

(15) $$Recall=\frac{TP}{TP+FN}.$$

The F1-score is used to define the prognosis, which is expressed as the weighted mean of precision and recall. A value of 1 defines good performance, whereas a value of 0 defines poor performance. F1-score in no way takes into account the TNs; it is computed as follows:

(16) $$F1-\mathrm{Measure}=\frac{2\times Precision\times Recall}{Precision+Recall}.$$

**Figure 5:**
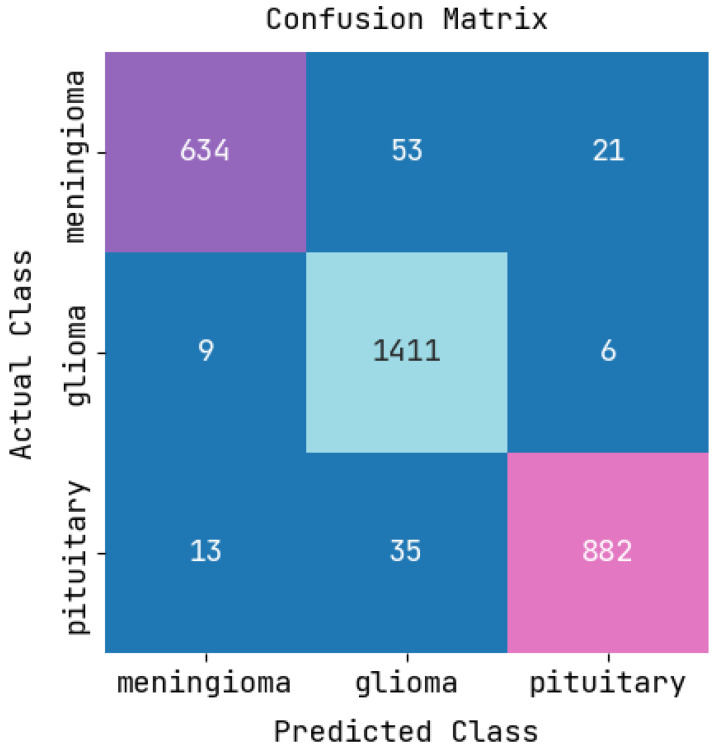
Confusion matrix.

Figure 5 exhibits the confusion matrix for features using a classifier testing model, in which the rows represent the predicted output, and columns represent the real data. The diagonal violet, blue, and pink colors represent tested networks that were correctly and incorrectly classified. The column on the right represents every predicted class, and the row at the bottom represents the performance of every actual class.

**Figure 6:**
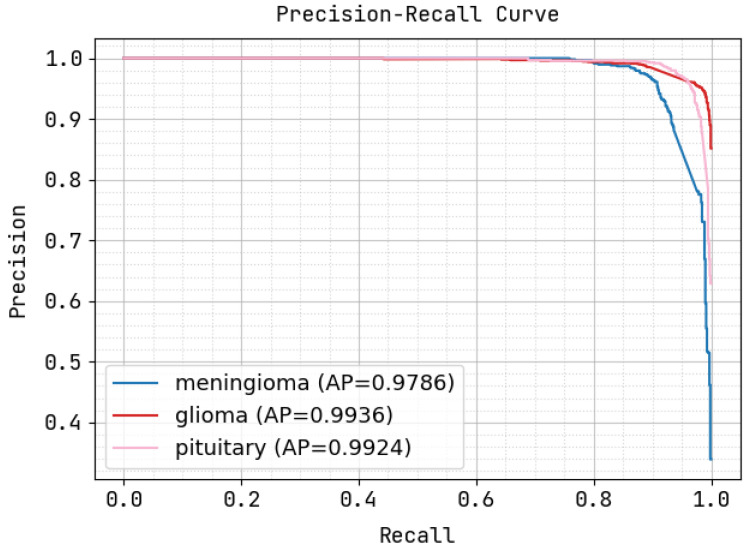
Precision–recall curve correlation.

Figure 6 illustrates the precision–recall curve, in which the X-axis exhibits the recall value, and the Y-axis exhibits the precision value. During this procedure, the proffered methodology attained an AP of 0.9785 for meningioma, 0.9936 for glioma, and 0.9924 for pituitary tumors, exhibiting the fine precision and recall of VGG16_CNN.

**Figure 7:**
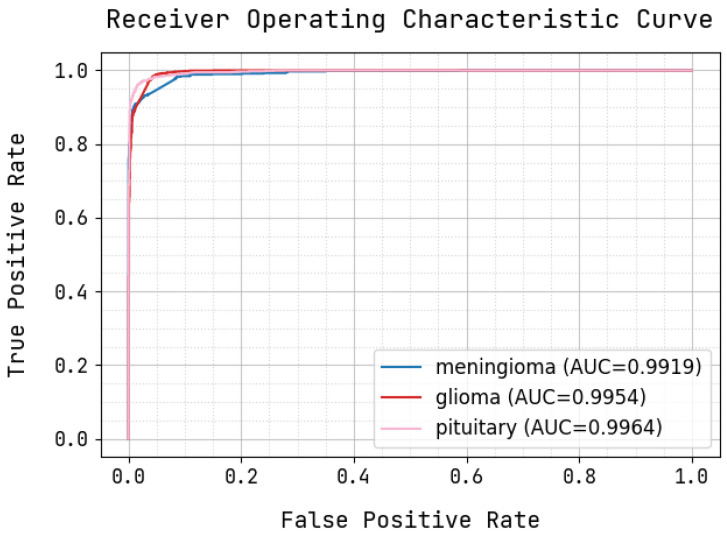
ROC curve correlation.

Figure 7 illustrates the precision–recall curve, in which the X-axis exhibits the FP rate, and the Y-axis exhibits the TP rate. During this procedure, the proffered methodology attained an AP of 0.9919 for meningioma, 0.9954 for glioma, and 0.9964 for pituitary tumors, exhibiting the fine precision and recall VGG16_CNN.

**Figure 8:**
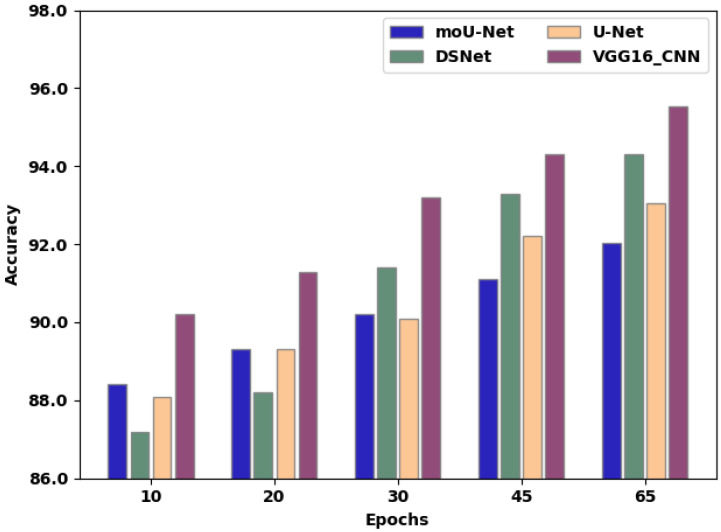
Accuracy comparison of existing techniques and the proposed method.

Table 2 presents the accuracy of existing techniques in comparison to the proposed method. Figure 8 exhibits an accuracy comparison of moU-Net, DSNet, U-Net, and the proffered VGG16_CNN methodology, in which the X-axis exhibits the quantity of epochs employed for assessment, and the Y-axis exhibits the accuracy values attained as a percentage. The moU-Net, DSNet, and U-Net methodologies attained accuracies of 92.02%, 94.3%, and 93.04%, respectively. The proffered VGG16_CNN methodology attained 93.74% accuracy, representing an improvement of 1.76%, 1.54%, and 0.7% compared to moU-Net, DSNet, and U-Net, respectively.

**Figure 9:**
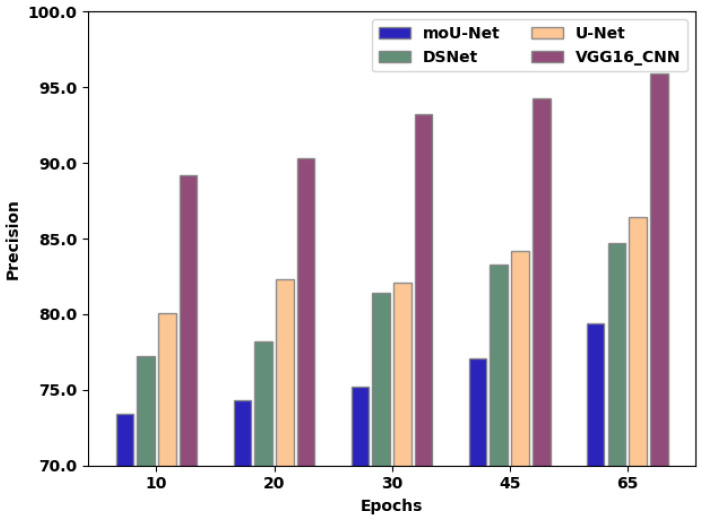
Precision correlation of the existing techniques and proposed method.

Table 3 presents the precision value of existing techniques and the proposed method. Figure 9 exhibits a precision comparison of moU-Net, DSNet, U-Net, and the proffered VGG16_CNN methodology, in which the X-axis exhibits the number of epochs employed for assessment, and the Y-axis exhibits the precision values attained as a percentage. The moU-Net, DSNet, and U-Net methodologies attained a precision of 79.4%, 84.72%, and 86.42%, respectively. The proffered VGG16_CNN methodology attained a precision of 92%, representing an increase of 13.4%, 8.72%, and 6.42% compared to moU-Net, DSNet, and U-Net, respectively.

**Figure 10:**
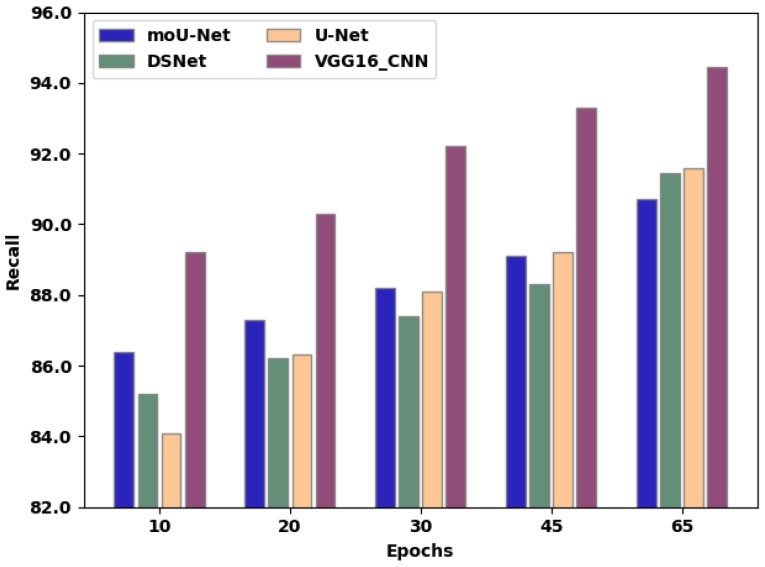
Recall comparison of the existing techniques and proposed methodology.

Table 4 presents the recall of existing techniques and the proposed method. Figure 10 exhibits the recall comparison of moU-Net, DSNet, U-Net, and the proffered VGG16_CNN methodology, in which the X-axis exhibits the number of epochs employed for assessment, and the Y-axis exhibits the recall values attained as a percentage. The moU-Net, DSNet, and U-Net methodologies attained a recall of 90.7%, 91.44%, and 91.58%, respectively. The proffered VGG16_CNN methodology attained a recall of 92.1%, representing an improvement of 2.6%, 1.34%, and 1.48% compared to moU-Net, DSNet, and U-Net, respectively.

**Figure 11:**
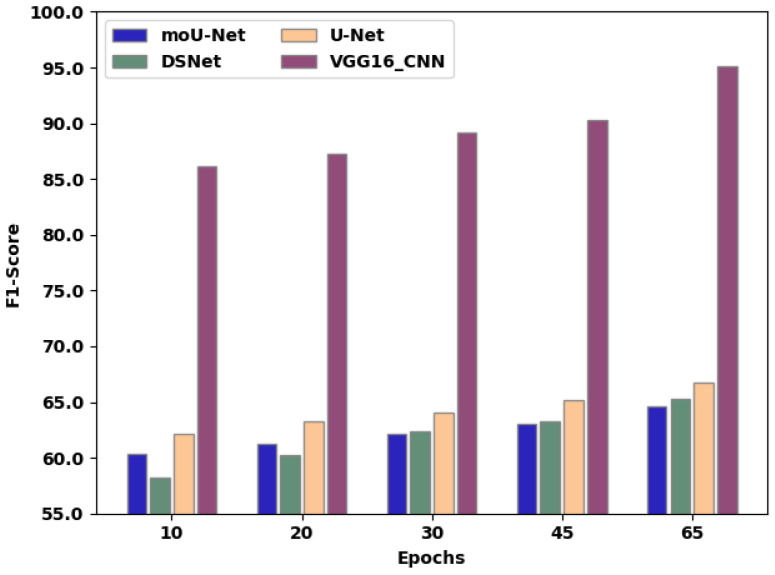
F1-score comparison of the existing techniques and proposed method.

Table 5 presents the F1-score of the existing techniques and proposed method. Figure 11 exhibits the F1-score comparison of moU-Net, DSNet, U-Net, and the proffered VGG16_CNN methodology, in which the X-axis exhibits the number of epochs employed for assessment, and the Y-axis exhibits the F1-score values attained as a percentage. The moU-Net, DSNet, and U-Net methodologies attained an F1-score of 64.62%, 65.24%, and 66.76%, respectively. The proffered VGG16_CNN methodology attained an F1-score of 67.08%, representing an improvement of 3.04%, 2.24%, and 2.3% compared to moU-Net, DSNet, and U-Net, respectively.

**Figure 12:**
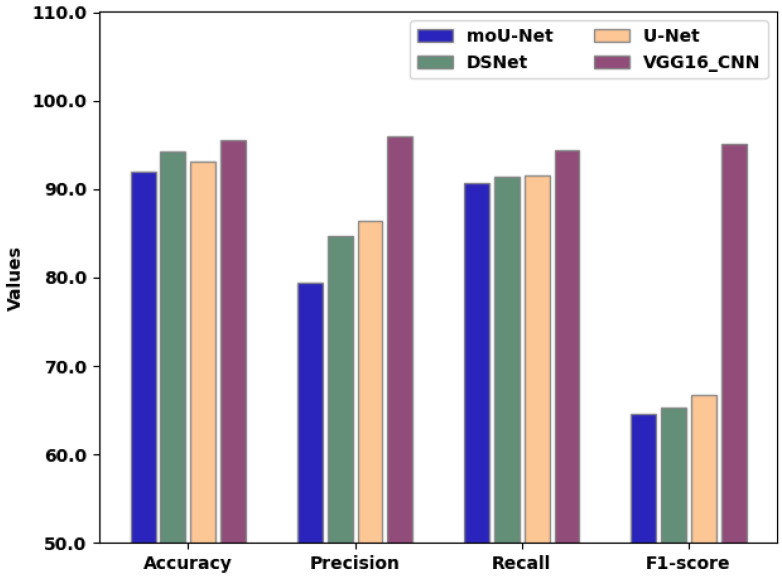
Overall comparison of existing techniques and the proposed method.

Table 6 presents the overall comparison of existing techniques and the proposed VGG16_CNN method, clearly revealing that the latter outperformed existing techniques in terms of the highest classified output. As shown in Figure 12, the training of features was performed to create a new feature array for training through the VGG16_CNN network.

**Figure 13:**
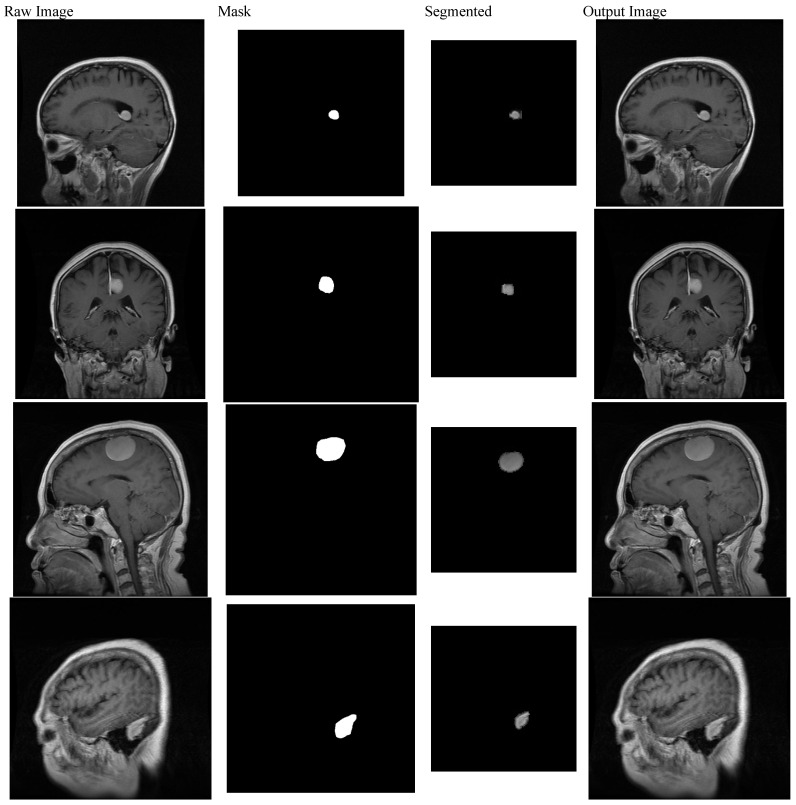
Various stages of the pre-processing (masking), segmentation, and output image of brain analysis.

Figure 13 depicts the overall flow of the proposed methodology at various stages of the brain metastasis approach.

Many brain imaging tools are available to cognitive neuroscientists, including positron emission tomography (PET), near-infrared spectroscopy (NIRS), magnetoencephalography (MEG), electroencephalography (EEG), and functional magnetic resonance imaging (fMRI). The steps involved in image preprocessing are reading, resizing, removing noise (denoising), segmentation, and morphology (smoothing edges), whereas image segmentation is a method of dividing a digital image into subgroups called image segments, thereby reducing the complexity of the image and enabling further processing or analysis of each image segment. For ABMS identification and segmentation, standard machine learning methodologies such as template matching, support vector machine, and AdaBoost are implemented. The moU-Net performs well for the segmentation of glioblastoma and low-rank glioma. The U-Net is marked by an encoder that outputs lower-grade portrayals of the input data (ID) and is associated with a decoder that rebuilds the correlated label map with skip connections between intermediate phases of the two units. The prognosis of patients with BMs is notably poor and ruins quality of life. The median survival of patients with BMs does not exceed 5 months in spite of the present therapeutic possibilities. However, the description of perfectly identified predictive subsets remains vital for the selection of a customized treatment plan. It remains significant to detect subsets of patients with suitable predictive attributes, which can then lead to the development of therapy targeting survival and an improved quality of life.

## 6. Conclusions

In this study, a DL-related classification method called VGG16_CNN was established for differentiating BMs by employing standard MR images. This method can be employed clinically by an expert radiologist to differentiate BMs, assisting in the classification of brain MRIs into meningiomas, gliomas, and pituitary tumors. Less extensive hardware specifications are needed, and large images (256 × 256) can be processed in an appropriate time. Furthermore, the VGG16_CNN classifier exhibits finer outcomes with respect to conventional classifiers such as moU-Net, DSNet, and U-Net. It can be noted that the proffered VGG16_CNN attained 93.74% accuracy, 92% precision, 92.1% recall, and 67.08% F1-score. Hence, the novel loss function can be computed by employing the weighted softmax function (WSF) for enhancing minute MtS diagnosis and for calibrating susceptibility and particularity.

## Figures and Tables

**Table 1:** VGG16_CNN’s weight and biases.

Name	Filter	Feature Map	Weights	Biases
Conv3-64	3 × 3 × 64	50 × 50 × 64	1728	65
Conv3-64	3 × 3 × 64	50 × 50 × 64	3456	56
Conv3-128	3 × 3 × 128	50 × 50 × 128	34,554	78
Conv3-256	3 × 3 × 256	50 × 50 × 256	34,579	88
Conv3-256	3 × 3 × 256	50 × 50 × 256	75,235	34
Conv3-512	3 × 3 × 512	12 × 12 × 512	57,561	36
Conv3-512	3 × 3 × 512	12 × 12 × 512	43,575	56
Atrous	3 × 3 × 512	3 × 3 × 512	34,591	78
Atrous	3 × 3 × 512	3 × 3 × 512	45,647	79
Conv 1 × 1	1 × 1 × 4	1 × 1 × 512	45,890	89
Conv 1 × 1	1 × 1 × 4	1 × 1 × 512	87,902	65

**Table 2:** Comparison of accuracy over various epochs between existing techniques and the proposed methodology.

Epochs	10	20	30	45	65
moU-Net	88.4	89.3	90.2	91.1	92.02
DSNet	87.2	88.2	91.4	93.3	94.3
U-Net	88.1	89.3	90.1	92.2	93.04
VGG16_CNN	90.2	91.3	93.2	94.3	95.53

**Table 3:** Comparison of precision over various epochs between existing techniques and proposed methodology.

Epochs	10	20	30	45	65
moU-Net	73.4	74.3	75.2	77.1	79.4
DSNet	77.2	78.2	81.4	83.3	84.72
U-Net	80.1	82.3	82.1	84.2	86.42
VGG16_CNN	89.2	90.3	93.2	94.3	95.94

**Table 4:** Comparison of recall over various epochs between existing techniques and proposed methodology.

Epochs	10	20	30	45	65
moU-Net	86.4	87.3	88.2	89.1	90.7
DSNet	85.2	86.2	87.4	88.3	91.44
U-Net	84.1	86.3	88.1	89.2	91.58
VGG16_CNN	89.2	90.3	92.2	93.3	94.44

**Table 5:** Comparison of F1-score over various epochs between existing techniques and proposed methodology.

Epochs	10	20	30	45	65
moU-Net	60.4	61.3	62.2	63.1	64.62
DSNet	58.2	60.2	62.4	63.3	65.24
U-Net	62.1	63.3	64.1	65.2	66.76
VGG16_CNN	86.2	87.3	89.2	90.3	95.12

**Table 6:** Comprehensive comparison between proposed and existing methodologies.

Criteria	moU-Net	DSNet	U-Net	VGG16_CNN
Accuracy (%)	92.02	94.3	93.04	95.53
Precision (%)	79.4	84.72	86.42	95.94
Recall (%)	90.7	91.44	91.58	94.44
F1-score (%)	64.62	65.24	66.76	95.12
